# Bayesian Alternation during Tactile Augmentation

**DOI:** 10.3389/fnbeh.2016.00187

**Published:** 2016-10-07

**Authors:** Caspar M. Goeke, Serena Planera, Holger Finger, Peter König

**Affiliations:** ^1^Institute of Cognitive Science, University of OsnabrückOsnabrück, Germany; ^2^Department of Neurophysiology and Pathophysiology, University Medical Center Hamburg-EppendorfHamburg, Germany

**Keywords:** sensory augmentation, tactile stimulation, vestibular system, multimodal integration, Bayesian alternation, subjective uncertainty

## Abstract

A large number of studies suggest that the integration of multisensory signals by humans is well-described by Bayesian principles. However, there are very few reports about cue combination between a native and an augmented sense. In particular, we asked the question whether adult participants are able to integrate an augmented sensory cue with existing native sensory information. Hence for the purpose of this study, we build a tactile augmentation device. Consequently, we compared different hypotheses of how untrained adult participants combine information from a native and an augmented sense. In a two-interval forced choice (2 IFC) task, while subjects were blindfolded and seated on a rotating platform, our sensory augmentation device translated information on whole body yaw rotation to tactile stimulation. Three conditions were realized: tactile stimulation only (augmented condition), rotation only (native condition), and both augmented and native information (bimodal condition). Participants had to choose one out of two consecutive rotations with higher angular rotation. For the analysis, we fitted the participants' responses with a probit model and calculated the just notable difference (JND). Then, we compared several models for predicting bimodal from unimodal responses. An objective Bayesian alternation model yielded a better prediction (χ_red_^2^ = 1.67) than the Bayesian integration model (χ_red_^2^ = 4.34). Slightly higher accuracy showed a non-Bayesian winner takes all (WTA) model (χ_red_^2^ = 1.64), which either used only native or only augmented values per subject for prediction. However, the performance of the Bayesian alternation model could be substantially improved (χ_red_^2^ = 1.09) utilizing subjective weights obtained by a questionnaire. As a result, the subjective Bayesian alternation model predicted bimodal performance most accurately among all tested models. These results suggest that information from augmented and existing sensory modalities in untrained humans is combined via a subjective Bayesian alternation process. Therefore, we conclude that behavior in our bimodal condition is explained better by top down-subjective weighting than by bottom-up weighting based upon objective cue reliability.

## Introduction

Humans sample information from their environment by many senses. In most circumstances (i.e., outside of the lab), behavior is not guided by a single modality but by a combination of several modalities. In the last decade many studies have shown that this process follows optimal Bayesian principles (Ernst and Bülthoff, 2004; Körding and Wolpert, 2004, 2006). A core concept of Bayesian integration is that perceptional noise (variance) is reduced in multimodal conditions, improving the precision of later decision processes. Many studies concentrated on the combination of visual and haptic cues. Ernst and Banks (2002) showed that visual and haptic information about object sizes are statistically optimally integrated. Extending this idea, Helbig and Ernst (2007) demonstrated optimal integration between vision and touch also for the shape of objects. Similarly, Reuschel and colleagues showed that visual and proprioceptive information are integrated in a statistically optimal manner for the perception of geometric trajectory (Reuschel et al., 2010). Moreover, several other combinations of senses have been investigated. Battaglia and colleagues found that visual and auditory information are optimally integrated in a spatial localization task (Battaglia et al., 2003). Frissen and colleagues reported optimal integration between proprioceptive and vestibular information for spatial updating (Frissen et al., 2011). Accordingly, Butler and colleagues argued that visual and vestibular signals are integrated in a Bayesian way for heading estimation (Butler et al., 2010). All in all, there is rich evidence that sensory information from different modalities is integrated following optimal Bayesian statistical principles.

While the concept of Bayesian optimal integration has been confirmed throughout several experimental paradigms, recent studies showing that integration happens only for redundant sensory information, i.e., both signals have to “describe” the same physical property. In this respect, Körding and colleagues demonstrated that the perceived causal relationship of two sensory signals is a prerequisite for sensory integration (Körding et al., 2007). Wozny et al. (2010) provided further evidence, sowing that the majority of their subjects used a probability matching strategy in a perceptual decision task. Furthermore, the integration of two sensory modalities requires a mapping between the two kinds of information. Mapping in this context refers to the cross-modal associations or correspondences of the sensory cues. For instance, there is a certain mapping of how it feels to hold an object in your hand and how it looks like. This association changes with the softness or weight of the object. Importantly, people can learn such a mapping, even if no prior coupling existed before. In particular, Ernst (2007) showed that subjects were able to optimally integrate visual cues (brightness) and haptic cues (stiffness). Similarly, Kaliuzhna and colleagues demonstrated that subjects integrated arbitrary co-occurring self-motion (vestibular) and visual cues (Kaliuzhna et al., 2015). Furthermore, Kuang and Zhang introduced a new visual-olfactory mapping. In their study the researchers linked two different smells to opposite movement directions in a dot movement discrimination task. After establishing such a pairing the presentation of the olfactory cues biased the perception of visual motion direction (Kuang and Zhang, 2014). For a detailed review regarding cross-model mappings also see Ernst (2006).

If new sensory-mappings are optimally integrated without or after very short training sessions one could ask the question, do humans innately integrate two co-occurring signals? If not, what would be possible alternatives? In 2008, Nardini and colleagues tested the concept of Bayesian optimal integration in a navigation task with three different age groups: children of 4–5 years of age, children of 7–8 years of age, and adults. Interestingly, they found that both groups of children did not integrate optimally between visual and proprioceptive cues but rather alternated between them. In contrast, adults performed the same task in a “*Bayesian optimal*” manner (Nardini et al., 2008). Similarly, Gori and colleagues reported that integration of vision and touch before 8 years of age is far from optimal (Gori et al., 2008). According to the authors, this was the case even when the dominating sense was made far less precise than the neglected sense. These results provide evidence that the capability of integrating information in a Bayesian optimal way requires several years of experience and is not an inherent property of our brain. More recently, Chen and McNamara tested how people integrate visual and self-motion cues during spatial navigation and found evidence for Bayesian Alternation even for some adult subjects (Chen and McNamara, 2014). Besides Bayesian Alternation, there is of course the possibility that people only use one cue and completely neglect the other. However, in such a case there is no cue combination, or multisensory processing at all. In fact also other recent studies provide evidence for cue alternation behavior (de Winkel et al., 2013, 2015; Adams, 2016). The general idea behind Bayesian cue alternation is that both cues are used for the task; however, they are never used at the same time. Instead, the subject switches between one and the other cue based on a Bayesian probability selection mechanism. Hence for each trial one or the other cue is selected while the probability for selecting one cue over the other is given by the respective relative weight for each cue. In summary, several studies in the last decade found evidence for cue alternation behavior. To our understanding this deviates from the majority of findings regarding Bayesian optimal integration and needs to be investigated in more detail.

The mechanisms that underlie the transition from cue alternation to cue integration are usually observable only in children or when sensory signals are explicitly manipulated (i.e., adding sensory noise). However, it is unclear what happens when adult subjects are equipped with a new sense (or an augmented sensory-like cue). Are we able to integrate such new information with the cues we receive from our native modalities or do we have to choose and rather alternate between the two (similar as children do)? In other words, it is most interesting to examine adults' performance when they are provided with a new, augmented sense which they have to combine with information from their existing (native) senses. We specifically ask the question: Is such a process similar or different to the ones observed in children? Throughout this paper we use the term “native modality” to refer to the information mediated by angular rotation through native sensors like the vestibular system, and augmented modality to information mediated by a sensory augmentation device, even as the subjects did not receive a formal training. Angular rotation in our setup was implemented by a rotaing platform on which the subjects were seated, while the augmented information was mediated via tactile stimulations (for details see the Section Experimental Paradigm). Although different combinations of sensory modalities are imaginable for sensory augmentation, tactile augmentation devices have preferentially been used in many research setups (Bach-y-Rita et al., 1969; van Erp and van Veen, 2003; Tsukada and Yasumura, 2004; Lindeman et al., 2005; Nagel et al., 2005). Besides academic research, the field of sensory augmentation recently also gained a lot of interest from industry. Many big companies lately introduced devices for augmented reality (e.g., Google Glasses, Microsoft Hololens, BMW Augmented Vision). However, while more products hit the market, there is a poor understanding of the underlying behavioral and neuronal mechanisms that reflect the process of combining the augmented and native senses.

Recently, Kaspar et al. (2014) performed a longitudinal study with a tactile augmentation device (the feelSpace belt) and reported that subjects developed an altered perception of space after a few weeks of training. Furthermore, it has been shown that tactile augmentation is particularly useful both in a visual search task (Wahn et al., 2016) as well as when participants are deprived of visual information (Faugloire and Lejeune, 2014). Hence, for the purpose of the current study, we built a rotating platform that was linked to a tactile augmentation device. In particular, we aimed to investigate whether people instantly combine an augmented tactile sense with vestibular information on whole body yaw rotation. Also none of the participants received any training with the augmentation device, as we intended to investigate the ability to instantaneously integrate augmented and native sensory information, rather than long-term training effects. As the main goal, we then compared prediction performance for the bimodal condition between a “winner takes all” (WTA) model and three more complex models: The Bayesian optimal integration model, and two types of Bayesian alternation models, one using objective measured weights and the other using subjective weights obtained via a questionnaire.

## Methods

### Tactile augmentation device and rotating platform

**Figure 1:**
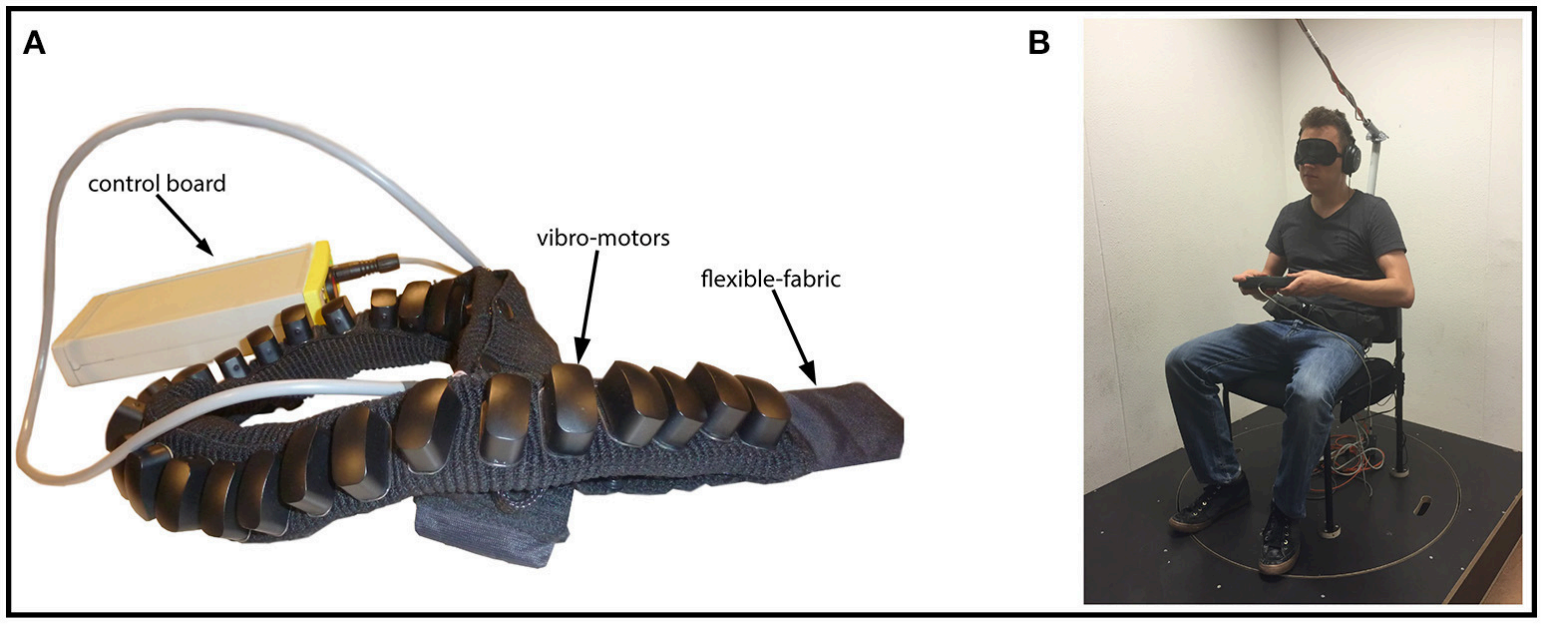
**Sensory augmentation device and rotating platform**. **(A)** Shows the tactile sensory augmentation device with its main components. **(B)** Illustrates the experimental setup. A participant is sitting on the chair fixed on the rotating platform and is wearing the tactile belt. He is additionally provided with an eye mask and headphones for noise cancelation. The participant is holding the response box in his hands.

Altogether we tested our participants in three conditions: rotating on the platform (native condition), receiving tactile vibrations around their waist (augmented condition), and both, rotating on the platform with simultaneous tactile vibrations (bimodal condition). Similarly to other setups in multimodal research, we employed a two-interval forced choice paradigm and tested participants in the two unimodal conditions (native or augmented) and the one bimodal condition (native plus augmented). Importantly, the tactile augmentation device and the rotating platform were precisely synchronized such that both signals provided redundant information. The tactile augmentation device (hereafter referred to as “tactile belt”) can, as the name suggests, be worn around the waist. An external computer controlled all 32 vibro-motors remotely via a serial port connection. The belt itself (Figure 1A) is made of a flexible fabric such that people with different abdominal sizes could wear it comfortably and the angular distance between two neighboring vibro-motors remained constant (~11.25°). During the whole experiment, all participants wore the belt just above their t-shirt or undershirt so that the elicited vibrations could be felt easily. When the tactile belt was switched on, at all times exactly one vibro-motor was active. For example, a rotation of 180° was accompanied by successive activation of half of the vibro-motors. Belt design and technology have been described in detail before (Nagel et al., 2005; Kärcher et al., 2012). To experimentally control angular rotation, we built a rotating platform with a chair fixed in the middle of it (Figure 1B). The platform could be remotely controlled, and precise parameters about angle and speed were adjusted on a trial-to-trial basis. Importantly, in the bimodal condition the vibration direction of the tactile belt was opposite to the rotation direction of the platform. In our setup, participants sat on the chair, were blindfolded, and wore headphones through which we played pink noise. Additionally, all participants held a response box with both hands, which was used for giving the required responses. They either pressed the left or the right button (indicating selection of the first or second rotation, respectively). A consecutive press on a button in the middle started the next trial.

### Experimental paradigm

Overall, the trial design was similar for all three experimental conditions. Importantly, the task and the information provided was identical, however, what varied between conditions was the type of sensory modality by which the information was provided. In the augmented (tactile-only) condition, only the belt vibration was activated. Here, participants had to judge angular differences purely based upon the successive tactile vibrations. In the native condition, only the platform rotated, so that the subjects had to rely only on rotational information. In the bimodal condition both the tactile belt and the platform were switched on synchronously and, therefore, subjects could use both sources of information. In all conditions, a trial consisted of two consecutive rotations (in the augmented condition only successive vibrations) with different angular sizes, with a one-second inter stimulus interval in between. The participants' task was to choose either the first or the second rotation (2 IFC task) depending on which of the two rotation angles was bigger. The participants had to press the left button to indicate that the first rotation was larger or the right button to indicate that the second rotation was larger. In fact, in half of the trials the first rotation was larger, and in the other half the second one was larger. After making their choice, the participants confirmed it by pressing the center button, whereupon which the next trial started immediately. Each trial consisted of a reference and a comparison stimulus. The reference stimulus was fixed at 146.25° (equivalent to a distance of 13 intervals between the vibro-motors) and kept constant throughout the whole experiment. The comparison stimulus varied in steps of 11.25°, the distance between two adjacent vibro-motors. The order of the reference and the comparison stimulus (i.e., which of the two was the first rotation) switched randomly on a trial-to-trial basis and was balanced overall for each subject and condition. We implemented 11 different combinations of rotation angles, ranging from five steps less than to five steps greater than the reference value (90°–202.5°), plus the condition in which both reference and comparison stimuli were identical. Each of these angle combinations was repeated 10 times in a random sequence within one modality condition. For all these trials, the speed was set constant to about 42°/s and the direction of the rotation was the same within a trial, but varied (in a balanced way) across trials. Additionally, we included 10 catch trials in each condition, for which we changed the speed between the two rotations (42° vs. 32°/s). Contrary to the “normal” trials, in the catch trials the shorter rotation (in time) was associated with a wider rotation (in rotational angle) and vice versa. This was used to evaluate how much each condition was influenced by cognitive strategies (e.g., counting time). Catch trials and normal trials were randomly intermixed. Altogether, each condition consisted of 120 trials that were recorded in a block. Participants were offered a chance to take a short break after each set of 40 trials and a larger break after each block (condition). The breaks within one block ranged from about 20 s to about 2 min depending on the subjects arousal level. The breaks between blocks ranged between 1 and 5 min also depending on the subject. Each session, including all three conditions, lasted for about two and a half hours. All participants came to the lab three times, and on each visit all three conditions were measured. The order of the conditions was balanced across subjects.

### Participants, data cleaning, and questionnaire

Overall, 30 subjects were recorded within a period of about 5 months. However, two subjects did not complete all sessions, which left us with 28 complete data sets (16 participants were females). The age grand average of these participants was 24.03 years (*SD* = 3.3 years). All of the participants were students at the University of Osnabrück and each subject received either 40 euros or eight “participant hours” (which are mandatory for psychology and cognitive science students) as reimbursement for their participation. Prior to the recordings all participants were informed about the purpose of the study and signed information and consent forms. Furthermore, ethical approval was obtained by the university institutional review board. Although, we tried to make the experience with the tactile belt as comparable as possible for all participants, subjective tactile sensation was arguably rather diverse. Hence, we removed the data of five participants for which the just noticeable difference (JND) could not be determined or could be determined only with very high uncertainty. These participants presumably had difficulties processing the tactile stimulus or misunderstood the task and the inclusion of their data would thereby decrease the plausibility of consecutive analysis. This procedure ensured that later analysis was based on robust measures. This left a total of 23 participants for the remaining analysis.

In addition to the two-interval forced choice task, all participants were required to fill out a questionnaire after each condition. The questionnaires were designed to find out how intuitive and difficult each condition was and how participants judged the reliability and relevance of the provided signals. Almost all questions were defined on a Likert scale (1–5) such that participants had to choose how much they agreed with a certain statement. The questionnaire was identical for the three sessions and most of the questions were also identical between conditions. For instance: “the task was difficult,” or “I was confident about my answers.” A few other Likert questions varied slightly between conditions, e.g., “The belt's signal was intuitively understandable” vs. “The rotation signal was intuitively understandable,” “The belt's signal was prominent in my perception” vs. “The rotation signal was prominent in my perception.” Besides the Likert based questions we also asked the participants to tell us which strategy they used from a fixed set of options (the complete questionnaire is provided in Section questionnaires in the Appendix of Supplementary Material). Completing a questionnaire after each condition and session all subjects filed out nine questionnaires in total.

### Analysis

The main analysis procedure can be summarized in three main steps: First, the JND, the Point of subjective Equality (PSE), and the uncertainty of the JND for each condition and subject were estimated using a probit model. Second, based on the observed unimodal JNDs, we calculated the predicted bimodal JND (individually for each subject) for all tested models. Third, using observed and predicted bimodal JNDs, we calculated the reduced chi-squared statistic (χ_red_^2^) for each model. The next two paragraphs will explain these steps in more detail. Furthermore, we describe the questionnaire analysis in Section Questionnaire Analysis.

#### Curve fitting

**Figure 2:**
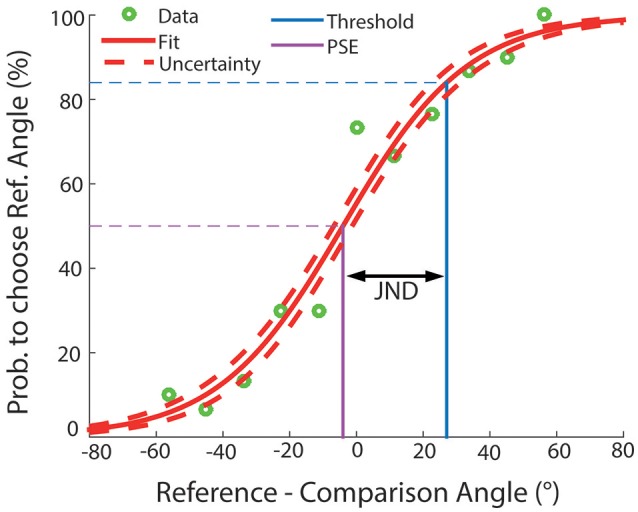
**Example logistic fit (native condition)**. The figure demonstrates the performance of one participant in the native condition as an example. The abscissa illustrates the difference between the two angular rotations (reference—comparison angle) in degrees. The ordinate indicates the probability to choose the reference angle. The green circles show the recorded behavioral data, the solid red curve shows the logistic fit, and the dashed red lines indicate the uncertainty of the fit. The magenta line depicts the Point of subjective Equality, while the blue line depicts the sensory threshold, at one standard deviation (84%) of the psychometric function. The distance between the PSE and the intersection of the blue line with the abscissa represents the JND.

In order to calculate the JND, we fitted for each subject and condition a GLM with a probit link to the behavioral data. The function is formalized in Equation (1), where β_1_ and β_2_ are the two (optimized) parameters of the model fit and the “norminv” function computes the inverse of the normal cumulative distribution function (cdf). We needed to invert Equation (1) in order to obtain the corresponding value of angular difference (x) for a specific performance level. Then, we used the asymptotic threshold of one standard deviation of a cumulative binominal distribution function (84%) as the corresponding angular difference of Equation (2) (y) and, consequently, calculated the JND. This gave us a direct measure of how precise each subject was able to distinguish the two angular stimuli from each other, separately for each condition. Next, we estimated the quality of the estimate of JNDs. Hence, we applied the error propagation method using the matrix formalism as described by Equation (3). Here, U_JND_ represents the uncertainty of the JND estimation, *V*_β_ is the covariance matrix of the betas, and J_i, j_(β), shown in Equation (4), is the Jacobian matrix. As an example plot Figure 2 demonstrates that most of the participants showed a behavior well-described by typical sigmoidal psychometric function.

(1) $$\begin{array}{rcl} \beta_{1}+\text{x}\cdot \beta_{2} & = & \text{norminv}\left(\text{y}\right) \end{array}$$

(2) $$\begin{array}{rcl} {\text{x}}_{\text{JND}}\left(\beta_{2}\right) & = & \frac{\text{norminv}\left({\text{y}}_{\text{threshold}}\right)}{\beta_{2}} \end{array}$$

(3) $$\begin{array}{rcl} {\text{U}}_{\text{JND}} & = & \sqrt{\text{diag}\left(\text{J}\left(\vec{\beta }\right){\text{V}}_{\beta }\cdot {\text{J}}^{\text{T}}\left(\vec{\beta }\right)\right)} \end{array}$$

(4) $$\begin{array}{rcl} {\text{J}}_{\text{i},\text{j}}\left(\beta_{1}{,\beta }_{2}\right) & = & \frac{\partial {\text{x}}_{\text{JND}}}{\partial \beta \text{j}}\left(\beta_{1}{,\beta }_{2}\right),\text{j}\in \left\{1,2\right\} \end{array}$$

#### Model comparison

The different models varied in their mathematical complexity for predicting bimodal performance. The simplest model was a static/intercept model which predicted always the same (mean) value for all subjects. The next one was a WTA model, which took either the native or the augmented JND (depending which of them was smaller) to predict bimodal JND. The Bayesian optimal integration model and the Bayesian alternation models were more complex. The Bayesian optimal integration model can be expressed as shown in Formula (5), while the Bayesian alternation model is described in Formula (6). μ illustrates the PSE of the psychometric function while P stands for the probability (i.e., relative weight) of each modality. In both Formulas (5) and (6), σ represents the JND, na is the abbreviation for the bimodal (native plus augmented) condition, n equals the native-only condition, and a stands for the augmented-only condition.

(5) $$\sigma_{\text{na}}^{2}=\frac{\sigma_{\text{n}}^{2}\cdot \sigma_{\text{a}}^{2}}{\sigma_{\text{n}}^{2}+\sigma_{\text{a}}^{2}}$$

(6) $$\begin{array}{rcl} \sigma_{\text{na}}^{2} & = & {\text{P}}_{\text{n}}\left(\mu_{\text{n}}^{2}+\sigma_{\text{n}}^{2}\right)+P_{\text{a}}\left(\mu_{\text{a}}^{2}+\sigma_{\text{a}}^{2}\right)-{\left({\text{P}}_{\text{n}}\cdot \mu_{\text{n}}+{\text{P}}_{\text{a}}\cdot \mu_{a}\right)}^{2} \end{array}$$

An interesting question regarding the Bayesian alternation model is how to determine the probabilities for the two unimodal modalities, P_n_ and P_a_. We decided to implement two different approaches. On the one hand, we used the observed objective (although subject specific) reliabilities such that the native probability could be formulated as described in Equation (7) and, analogously, the augmented probability as in Equation (8).

(7) $$\begin{array}{rcl} P_{\text{n}} & = & \frac{\frac{1}{\sigma_{\text{n}}^{2}}}{\frac{1}{\sigma_{\text{n}}^{2}}+\frac{1}{\sigma_{\text{a}}^{2}}} \end{array}$$

(8) $$\begin{array}{rcl} {\text{P}}_{\text{a}} & = & \frac{\frac{1}{\sigma_{\text{a}}^{2}}}{\frac{1}{\sigma_{\text{a}}^{2}}+\frac{1}{\sigma_{\text{n}}^{2}}} \end{array}$$

On the other hand, we calculated native and augmented weights on the basis of the individual questionnaire responses. For this procedure, we selected the following eight performance relevant questions of the native and the augmented parts of the questionnaire (“I have done similar tasks before,” “The task was intuitive,” “The task was difficult,” “I think I performed well in the task,” “I was confident about my answers,” “I felt comfortable with the task,” “The belt / the rotation gave me relevant information to solve the task,” “The belt/the rotation signal was prominent in my perception”). As all these questions were answered on a Likert scale, we could directly apply mathematical operations on them. First, we averaged the responses of the three different sessions separately for each question and then subtracted answers relating to the native condition from those relating to the augmented condition. As a result, for each question we knew whether the augmented or the native task was more intuitive, difficult, and so on (positive numbers indicated higher agreement in the augmented task, negative numbers indicated higher agreement in the native task). In order to combine the responses of all questions, we applied a principal component analysis resulting in eight different components. To further reduce dimensionality and to calculate subjective weights we then considered only the first component for further processing. Through this procedure we reduced all questionnaire responses to one scalar per subject. Finally, we normalized this number to the range of zero to one using a logistic function. These values were then used as augmented weights P_a_. The native weights P_n_ were then defined as the inverse 1 − P_a_. Although the complexity in terms of the mathematical expression varied between the models, we want to emphasize that we did not optimize free parameters for any model. In summary, we optimized the estimation for the observed JND, but we did not fit/improve unimodal to bimodal prediction performance by adjusting model parameters. Hence the amount of free parameters (*k*) was zero, and consequently the degrees of freedom (*v* = 22 = *N* − *k* − 1) were constant throughout all investigated models.

#### Questionnaire analysis

The main goal of the questionnaire analysis was to create a deeper understanding of the quantitative measurements. Therefore, we first looked at single questions in the unimodal parts and examined possible differences between the augmented and native ratings. Second, we analyzed the categorical responses about strategy use in all conditions in order to get a better estimate of how each participant subjectively approached the task. Here, all subjects had to choose one out of the following options: (a) tactile cue only, (b) rotation only, (c) combination of both cues, (d) counting time, (e) visualization, (f) random guessing, (g) other. We decided to focus only on these two analyses in order to keep a clear structure.

## Results

### Control statistics

**Figure 3:**
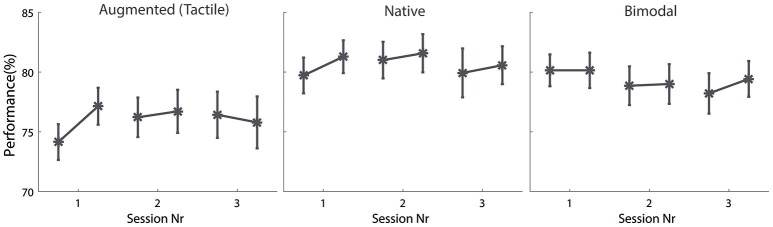
**Investigation of learning effects**. The abscissa divides the data of the three different sessions and the data of each session between the first half and second half of the block, separated by condition. The ordinate indicates the performance as a percentage. The error bars illustrate the average performance with the error bars representing the standard error of the mean.

First of all, we aimed to investigate whether the subjects exhibited non-stationarities within and/or between sessions, for example, in the form of learning or fatigue effects. Hence, we split the data for each condition and session into the first and second “block-half” and performed three separate repeated measures analyses of variance (one for each condition) with session and block-half as independent (repeated) variables and the number of correct responses as dependent variable. Catch trials were not considered in this analysis. However, as shown in Figure 3, neither session nor block-half nor the interaction of both factors revealed a significant influence in any of the three conditions (please find the analysis of variance tables in the Appendix in Supplementary Material). This indicates that the subjects' performances were constant within and between sessions. As the data did not reveal any indication of learning or fatigue effects, we then collapsed the data over all three sessions and calculated the amount of correct responses separately for each angle combination.

### Comparing conditions

**Figure 4:**
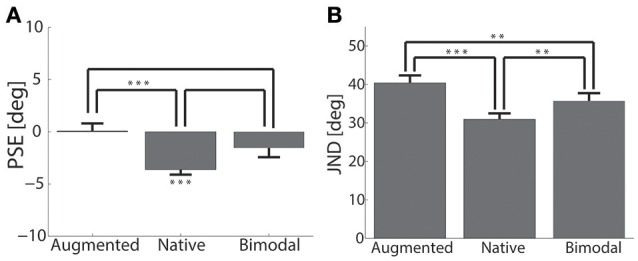
**Comparing conditions**. **(A)** Shows the PSE (on the ordinate) separately for the three different conditions on the abscissa as a mean over subjects. The asterisks below indicate the significance level for the difference of the SPE to zero. The asterisks above show the significance level for the comparisons between conditions. **(B)** Shows the JND again separately for the three different conditions on the abscissa and as a mean over subjects. The asterisks illustrate the level of significant differences between conditions.

After calculating the JNDs and PSEs (see Section Curve Fitting), we compared both measures between experimental conditions. Figure 4A displays the results for the PSE. First we applied separate *t*-tests for each condition to test whether the PSE was different from zero. While for the augmented condition [*t*_(22)_ = 0.109, *p* = 0.914] and the bimodal condition [*t*_(22)_ = −1.726, *p* = 0.098] the PSE was not significantly different from zero, the native condition revealed a significant difference [*t*_(22)_ = −7.422, *p* = < 0.001]. Furthermore, we analyzed the PSE using a repeated measures ANOVA for the factor condition, which revealed a significant effect [*F*_(2, 44)_ = 7.976, *p* = 0.001, partial η^2^ = 0.266]. *Post-hoc* comparisons confirmed a significant difference between the native and the augmented PSE (*p* = 0.001), but no significant difference between the augmented vs. bimodal PSE (*p* = 0.101). The native vs. bimodal contrast was borderline non-significant (*p* = 0.051). Importantly, as a measure of the subjects' performance we analyzed the JND; Figure 4B illustrates these results. For the statistical analysis of the JND we also applied a repeated measure ANOVA. The result revealed a main effect of condition [*F*_(2, 44)_ = 17.869, *p* < 0.001, partial η^2^ = 0.448]. *Post-hoc* pair-wise comparisons confirmed that the JND in the augmented condition was higher than in the native (*p* < 0.001) and the bimodal (*p* = 0.010) conditions. The JND in the bimodal condition was, in turn, higher than in the native condition (*p* = 0.003). Hence, the native condition resulted in the best performance, followed by the bimodal condition; the augmented (tactile) performance was the worst. This rather compelling result indicates that native and augmented sensory modalities were not combined in a “Bayesian optimal way,” as this would require that the bimodal JND is less or equal than in either single modality. This raises the question of alternative models to be compared in the following investigation. As it is the gold standard in many studies on multisensory integration, we kept the Bayesian integration model in the model comparison procedure and compared it to several alternatives as described in the next paragraph.

### Model comparison

The main goal in our study was to determine the cognitive mechanism that underlies the combination of the augmented and native sensory cues provided. To address this central question of the study, we compared the five different models in their accuracy to predict the bimodal JND given the unimodal JNDs [Intercept, Winner Take All (WTA) optimal Bayesian integration, objective Bayesian alternation, subjective Bayesian alternation]. In particular, we combined the model prediction with the uncertainty measurement to calculate the reduced chi-squared value (χ_red_^2^), as shown in Formula (9).

(9) $$\chi_{\text{red}}^{2}=\frac{1}{\text{v}}\sum_{\text{k }=\;1}^{\text{n}}\frac{{\left({\text{JND}}_{\text{observed}}^{2}{-\text{JND}}_{\text{estimated}}^{2}\right)}^{2}}{{\left({\text{JND}}_{\text{uncertainty}}^{2}\right)}^{2}}$$

**Figure 5:**
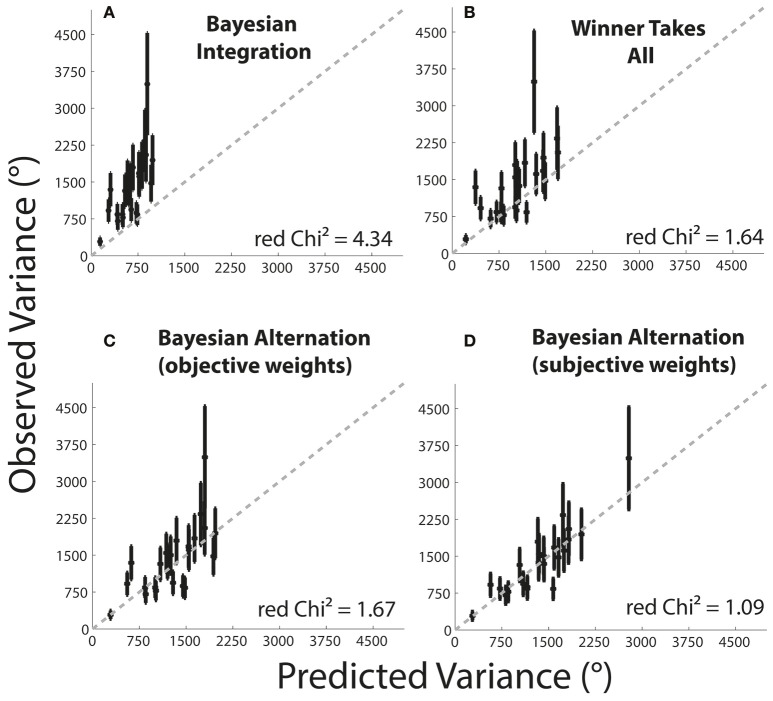
**Model comparison**. The abscissa shows the predicted squared JND; the ordinate shows the observed squared JND in the bimodal condition. Each black dot shows the predicted vs. the observed value for one subject. The error bars around the black dots illustrate the uncertainty of the observed bimodal values. The gray dashed diagonal line represents the ideal prediction. The resulting χ_red_^2^ is plotted for each model. **(A)** Bayesian integration. **(B)** Winner takes all. **(C)** Bayesian alteration (objective weights). **(D)** Bayesian alteration (subjective weights).

This gave us a measure of how much variance each model could explain compared to the optimum (χ_red_^2^ = 1), when all structure is explained and the residual variance is due to noise only. Our results show that the intercept model is a poor fit for the data (χ_red_^2^ = 10.95) and leaves a lot of variance to be explained. Figure 5 summarizes the result for the other four models of interest. Although the Bayesian integration model (Figure 5A) is clearly a better model than the intercept model, it also leaves quite some variance to be explained (χ_red_^2^ = 4.34). While the objective Bayesian alternation model outperformed the integration model (Figure 5C, χ_red_^2^ = 1.67), the WTA model predicted bimodal behavior even slightly better (Figure 5B, χ_red_^2^ = 1.64). However, using subjective weights for the Bayesian alternation model, prediction performance could be significantly improved such that it had the highest prediction rate and lowest residual variance among all tested models (Figure 5D, χ_red_^2^ = 1.09). In fact, the χ_red_^2^ of the subjective Bayesian alternation model is very close to the optimum of χ_red_^2^ = 1.00.

### Subjective vs. measured reliabilities

**Figure 6:**
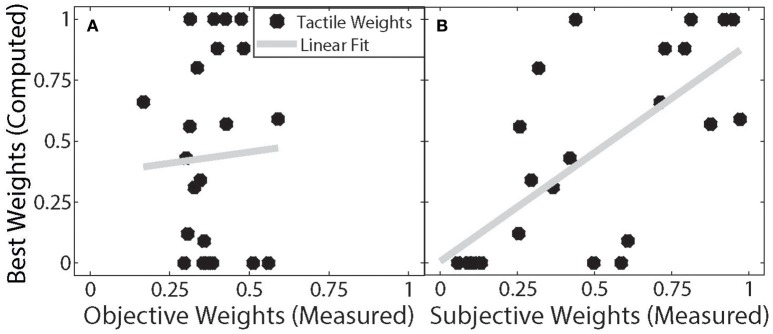
**Correlation of measured and computed weights**. The ordinate shows the individual tactile weights that lead to the best possible model fit of the Bayesian alternation model. **(A)** Shows the correlation of the best weights and the actual measured weights based on measured reliability. **(B)** Shows the correlation of these estimated weights and the weights calculated from the questionnaire data. Each black dot represents one participant. The gray lines show the least square linear fit to the data.

To better understand the differences between the two types of Bayesian alternation models, using different unimodal weights *Pn* and *Pa*, we implemented an optimization procedure to find the weights that yielded the optimal prediction accuracy for each subject (referred to as the “optimal predicted weights”). That is, we did not investigate how subjects could perform optimally, but which type of weights for native and augmented modality (per subject) would optimally explain the data as they were observed. The two weights that were used in the model comparison procedure (objective and subjective) were then analyzed against these optimal predicted weights using a linear regression. As shown in Figure 6A, the comparison of the optimal predicted weights and the objective (based on unimodal performance) weights were uncorrelated [*r*_(22)_ = 0.0008, *p* = 0.887]. In constrast, the subjective (questionnaire-based) weights showed a strong and significant correlation with the optimal predicted weights [*r*_(22)_ = 0.4782, *p* < 0.001, Figure 6B]. This result indicates that compared to the objective/measured reliabilities, the subjective evaluations (weights) better captured the intersubject variability of cue preferences.

### Strategy assessment

**Figure 7:**
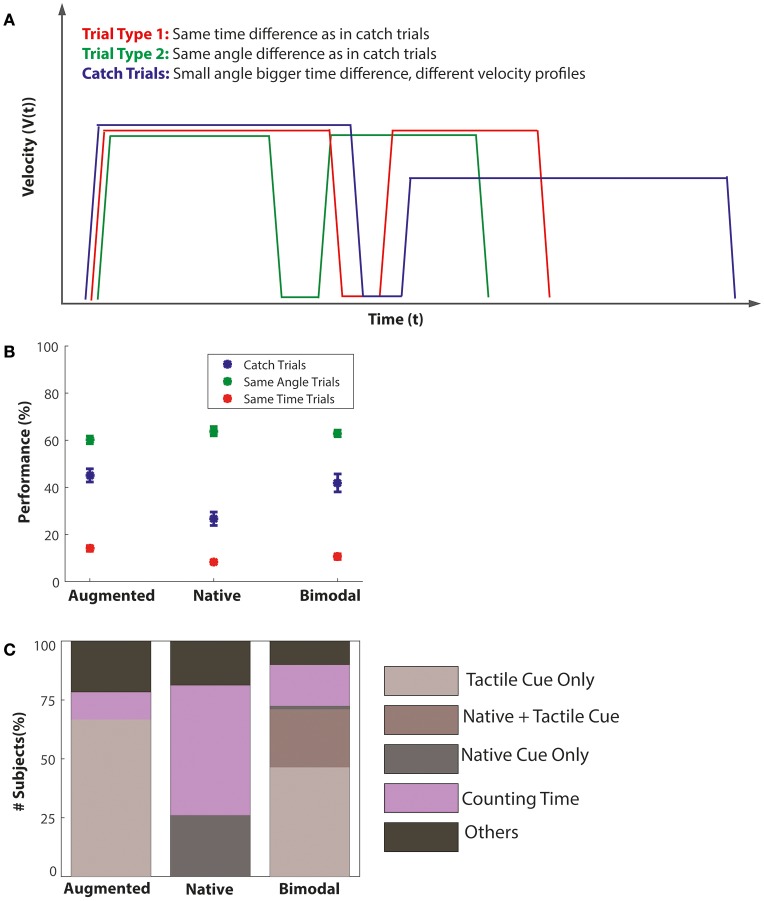
**Strategy assessment**. **(A)** Displays the velocity profiles of the different trial types that were analyzed in the catch trial analysis. The green line represents a “hard trial,” with small angular difference. The red line shows an easy trial with large angle (and time difference). The blue line illustrates the catch trial with inverted angle-time difference (the shorter rotation took more time). **(B)** Displays the result of the catch trial analysis. The abscissa separates the three experimental conditions. The ordinate illustrates average performance across subjects. The blue error bars show the performance in the catch trials, the red error bar shows the performance for the easy trials (same time difference as catch trials), and the green error bars show the performance for the hard trials (same angular difference as the catch trials). **(C)** Shows the result of the questionnaire analysis regarding subjective strategy use. The abscissa again separates the three experimental conditions and the ordinate indicates the proportion of subjects using a particular self-assessed strategy. The different strategy types are color coded and labeled.

All participants were deprived of visual information and could not use auditory information due to the pink noise played on the earphones. As angular rotation activates the semicircular canals, which are part of the vestibular system, the main sensory input here was the vestibular modality (for that reason we use the term native modality). However, processing angular rotation without visual information might have been a rather unusual experience for many of our participants. In theory, participants could therefore have also used some more cognitive strategies like counting time or visualizing images. In order to address this question, we analyzed the catch trials and the subjective questionnaire data (directly asking for the strategy employed). For the catch trial analysis, as shown in Figure 7A, we compared the performance in three types of trials: first, the performance in the catch trials itself, which had reversed angular-time differences; second, the performance in trials with the same angular difference as in the catch trials (11.25°) but a much shorter time difference (~250 ms); and third, the performance in trials with 45° angular difference, as they were most similar in the time domain to the catch trials (~1100 ms difference), but very different in the angle domain. Figure 7B shows that in the augmented tactile task, performance in the catch trials (blue) was more similar to the trials with the same angle difference (green), compared to the trials with the same time difference (red). This supports the view that subjects used angular but not time information in this condition. However, this pattern was reversed in the native task, such that catch trial performance in the native task was more similar to same-time trials. Hence, the native task was clearly influenced by time (counting) information. The bimodal task performance was again in between these two, with a trend toward the angle-based trials, supporting the idea that signal/strategy usage alternated on a trial-to-trial basis. Figure 7C illustrates the results of the subjective strategy assesment. In the respective (bimodal) question “Which strategy did you use to solve the task?” all subjects had to choose one out of following the options: belt only, rotation only, combination of belt and rotation, time counting, visual imagination, random guessing, other strategy. As the last three options (visual imagination, random guessing, and other strategy) were chosen only rarely (each < 8%) we summarized them to “other strategies.” Overall, the results of the questionnaire analysis are in line with the catch trial analysis. Subjects reported to have used the time information in only 8 out of 69 sessions for the augmented tactile task. In the bimodal condition, subjects reported that counting time was their preferred strategy in 12 out of 69 sessions. Again, the native task showed a reversed picture. Here, participants reported that they were counting time in 38 out of 69 sessions (23 subjects ^*^ 3 sessions = 69 total sessions). Overall, both questionnaire and catch results indicate that most subjects relied on cue processing in the augmented and bimodal tasks and suggest that counting time and other cognitive strategies played a major role in the native condition.

### Relevance and dominance of the signals

**Figure 8:**
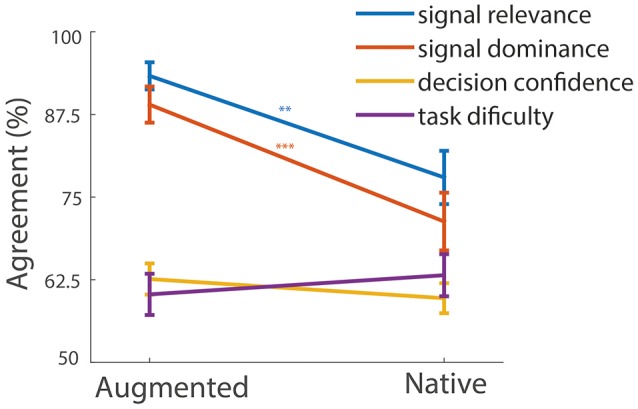
**Signal perception**. The abscissa separates the tactile and the vestibular condition. The ordinate indicates the level of agreement for a certain question. The error bars are standard errors of the mean. The asterisks indicate the level of significant difference between the two conditions.

As a final step, we investigated differences between augmented and tactile questionnaires for individual questions. Most questions did not reveal interesting or significant differences between the augmented condition and the native condition. Figure 8 contrasts the agreement (mean over subjects) for the following four questions between the native and the augmented condition: 1. “The belt/the rotation gave me relevant information to solve the task,” 2. “The belt/the rotation signal was prominent in my perception,” 3. “The task was intuitive,” 4. “The task was difficult.” The analysis showed that the native task was perceived as more difficult; however, the difference from the augmented task was not significant [*t*_(22)_ = −0.91, *p* = 0.373]. In line with this observation, subjective decision confidence was higher in the augmented condition, but again failed to reach significance [*t*_(22)_ = 1.21, *p* = 0.2402]. However, two other questions showed clear effects; the first was signal relevance, the other was probing signal dominance. Participants judged the tactile belt as providing information with higher task relevance compared to the angular rotation of the platform [*t*_(22)_ = 3.34, *p* = 0.0030]. Similarly, the belt was rated to be perceptually more dominating [*t*_(22)_ = 4.36, *p* = 0.0002] than the native information.

## Discussion

### Summary

We tested whether untrained adult participants are able to use augmented tactile information in a two-interval forced choice task and examined how such augmented information is combined with information from native senses. Psychometric data and consecutive statistical analysis show that all subjects were able to solve the task using only the tactile information from the augmented sense. Hence, even without prior information or experience the participants were able to use the supplied augmented tactile information for the current task. The model comparison demonstrated that the subjective Bayesian alternation model had the highest prediction performance. This model reflects the idea that on each trial a subject is using one or the other sensory signal provided, caused by a (probability based) Bayesian selection mechanism. This finding is in line with earlier findings on Bayesian Alternation observed in children (Gori et al., 2008; Nardini et al., 2008). However, a more precise look at the data revealed that about half of the subjects strongly preferred one of the cues (native or augmented) while the other half used both cues more evenly. As a result, the respective weights for subjects with such strong preferences are matching a simple winner take all (WTA) strategy where these weights are set to one and zero, respectively. Although such behavior could be described with a much simpler WTA model, the Bayesian alternation model yields clearly higher prediction performance on a group level. This was due to the fact that the other half of the subjects alternated between both cues more often so that this behavior was better captured with the Bayesian alternation model. Altogether one can say that the spectrum of cue preferences was rather continuous between subjects. Some subjects preferred the augmented cue, some others preferred the native cue, and again many others were in-between these extremes. To put it differently, one can consider the subjective Bayesian alternation model as an extension of a WTA strategy. In particular it is more flexible as it allows to alternate signal usage on each trial compared to each subject (but doesn't require it). Moreover, we would like to point out that this does not involve fitting free parameters, but is purely based on observed unimodal performance and questionnaire data. The difference in prediction performance between objective and subjective alternation model is of further interest. Hereby, we demonstrated that a combination of qualitative and quantitative data represents an interesting and advantageous method in the field of multimodal research.

### Potential shortcomings of the study

One concern in the current study is related to the native modality condition. Although sensory input was provided only to the vestibular system, roughly half of the participants presumably involved a cognitive strategy such as counting time. However, the vestibular system necessarily has to integrate information over time and cannot provide an absolute reference. Hence, it would not be reasonable to assume that time information does not play any role for the vestibular system. From this perspective, we argue that the vestibular sense is to some extent a “time-angle integrator.” This idea was also discussed in a study by Berthoz et al. (1995). Furthermore, the catch trials were the hardest trials to solve with an angle-based strategy, as they not only had a very small angular difference, but also particularly long rotations (202.25° vs. 191°). Hence, subjects who aimed to use angular information in catch trials basically had to guess. As a result, some subjects might have used the counting strategy mostly in the catch trials in order to avoid guessing. Grondin and colleagues showed that humans benefit from a time counting strategy especially when judging intervals longer than 2.5 s (Grondin et al., 1999). Similarly, Clément and Droit-Volet showed that adults temporal sensitivity increases with explicit time counting, while this is not the case for children (Clément and Droit-Volet, 2006). In fact, fewer participants subjectively claimed in the questionnaire to have used a time-counting strategy as the catch trial analysis suggested. In conclusion, we argue that the native condition was influenced by both vestibular signals as well as higher cognitive strategies, in particular counting time. However, cognitive strategies did not make a major contribution in the augmented (tactile) or in the bimodal condition. Altogether, the investigation of signal/strategy use supports the idea that the majority of subjects used a subjective Bayesian alternation process to combine both sensory stimuli.

A second issue concerns the tactile belt and differences among individuals. Although, we tested the augmentation device before each session, some subjects reported that they sometimes did not properly feel the vibration. Differences in waist size, position of the vibro-motors, undershirt material, and the participant's ability to differentiate tactile stimulations might have altered perception of the tactile sensation. Due to technical limitations, in some cases one or the other vibro-motor might also have vibrated less strongly than others. To counteract these issues, we removed participants for whom we could not reliably estimate the psychometric performance (JND) that was later needed in the model comparison and other analysis (as described in the Method Section). Our results clearly show that most subjects follow a subjective Bayesian alternation strategy for the combination of native and augmented sensory cues. However, differences among individuals were strong enough that our conclusion is reasonable for the majority of the subjects, but not for each and every individual. Weights that led to an optimal prediction showed that many participants strongly preferred the augmented cue, while other subjects had a clear bias in favor of the native signal, and still others lay in between these two extremes. Importantly, the subjective questionnaire data helped to better understand those individual differences in performance measures and time-counting strategies. All in all, subjective and objective measurements nicely match and complement each other and hereby create a more complete picture of the reported findings.

### Integration vs. alternation

Many studies suggest that human multimodal processing involves “optimal integration.” Without arguing against such an overwhelming and high quality amount of empirical evidence, our findings qualify this statement to some extent. In fact, a closer look into the literature reveals that several studies reported deviations from the “standard” Bayesian integration model. One of best examples were reported by Nardini and colleagues as well as Gori and colleagues, both providing clear evidence that optimal integration is not present in children until the age of 8 years (Gori et al., 2008; Nardini et al., 2008). Most recently, Adams compared different integration models with an audio-visual temporal judgment task and similarly reported that older participants employed a partial integration strategy while younger participants (<8 years) did not integrate, but instead switched between the two sensory signals provided (Adams, 2016). Besides research with infants, there is evidence that under certain circumstances even adults do not integrate, but instead alternate between two sensory cues. In particular, de Winkel et al. (2013, 2015) performed a visual-vestibular cue combination task in which adult participants were rotated around the yaw axis, given either additional visual information or not. Most interestingly, the authors reported that only about half of the participants behaved in congruence with the Bayesian integration model, while the others most likely alternated in the usage between the two cues (de Winkel et al., 2013, 2015). One of the possible explanations for both, our results as well the experiments from de Winkel et al. (2013, 2015) would be that the two sensory signals were not perceived to have a common cause (Körding et al., 2007), although they were supplying redundant information. As Ernst (2007) and Kaliuzhna et al. (2015) showed, it is possible that humans (directly) integrate two arbitrary associated sensory signals. However, combining rotational information and (augmented) tactile stimulation might require a more complex mapping than the visual-haptic associations used in these studies. An interesting idea for a follow up study of our paradigm might be to explicitly force the integration, or at least comparison of both cues. In such a scenario the information in the first interval could be provided tactilely, while for the second interval the information would be displayed via the platforms rotation (or vice versa). How well participants can solve such a task needs to be addressed in future research.

### Multisensory learning

The comparison of prediction performance between the Bayesian integration model and the Bayesian alternation model showed that participants in our study most likely alternated between using augmented and native information. Research with infants has provided evidence that optimal integration of sensory cues is not a native mechanism, but instead has to be acquired (Gori et al., 2008; Nardini et al., 2008). Moving to the other side of the age spectrum, Bates and Wolbers recently showed that the combination of visual and self-motion cues becomes less than optimal with age. The authors attribute this observation to neural degeneration in entorhinal and hippocampal regions (Bates and Wolbers, 2014). Accordingly, neural degeneration and atrophy were shown to increase with age (Dickerson et al., 2001). In general, recent studies have shown that multisensory influences arise relatively early and by a variety of mechanisms (Driver and Noesselt, 2008). In a review from 2008, Stein and Stanford argued that many multisensory neurons exist in the superior colliculus. Explicitly they showed that this region combines visual, auditory, and somato-sensory input to control eye and head movements (Stein and Stanford, 2008). Burnett and colleagues tested this assumption by lesioning cats superior colliculus and conclude that damage to this area directly causes a loss of multisensory neurons which again led to a decrease of multisensory behavior (Burnett et al., 2004, 2007). Hence, one can conclude that optimal cue integration is experience dependent and relies on intact neural structures.

While children presumably take a couple of years to successfully integrate information originating from two native modalities, it has been unclear until now how such a process is established with an augmented sense in adults. Here, we provide the first evidence that the majority of adult participants combine augmented and innate sensory modalities using a subjective Bayesian alternation strategy. However, we speculate that intensive training with the sensory augmentation device could lead to a shift in the cue combination strategy. Specifically, over time Bayesian cue alternation might be replaced by optimal Bayesian cue integration, which might be associated to casual inference mechanisms described by Körding et al. (2007). In such a scenario the augmented tactile stimulation would improve overall performance. In line with this idea, several studies showed that training alters the individual reliabilities in a cue combination paradigm (Jacobs and Fine, 1999; Atkins et al., 2001). Furthermore, Shams and Seitz (2008) provided striking evidence that multimodal learning is more effective than unimodal learning. Hence, as a next step we plan to conduct a longitudinal study and investigate how training with the augmentation device will change cue combination strategies in adults.

### Cue combination and attention

There has been a long debate whether attentional resources share a common reservoir (Jolicoeur, 1999; Arnell and Larson, 2002) or whether each modality has its own attentional resources (Potter et al., 1998; Talsma et al., 2006; Martens et al., 2010; Wahn and König, 2015a,b, 2016). In fact, attention might have played an important role also in our study. Subjects reported that the tactile stimulation dominated their perception to a significantly stronger degree than did the angular rotation. Hence the participants' attention was driven toward the tactile stimulation. The observed Bayesian alternation process can therefore also be understood as an attentional mechanism. In this view, both cues rivaled for attentional focus such that it switched on a trial-to-trial basis, with a probability that was based on subjective reliability. In conclusion, our results support the idea of a shared reservoir of attention for native and augmented sensory cues.

A second issue is concerned with the attentional load. Several studies suggested that attentional or perceptual load modulates multisensory integration (Alsius et al., 2005; Mozolic et al., 2008; Klemen et al., 2009). Oppositely, Helbig and Ernst (2008) demonstrated that haptic cue weighting is independent of modality-specific attention. Similarly, Wahn and König (2015a,b) showed the existence of optimal integration between visuotactile and audiotactile cues even under high attentional load. In our study, attentional load was not modulated; however, considering the fact that cognitive strategies such as counting time played a major role only for the native but not for the augmented condition, future investigations with varying attentional load might reveal interesting new insights.

### Subjective vs. objective measurements of reliability

Our results demonstrate that objective measured reliability was higher in the native condition compared to the augmented condition. Similarly, Fetsch and colleagues demonstrated that vestibular cues are overweighted in low-reliability conditions (Fetsch et al., 2009, 2011). However, our participants reported (subjectively) that the tactile belt provided the more relevant information for the task, and the confidence ratings were slightly higher in the tactile condition. This discrepancy between the subjective awareness of a signal's reliability and its objective reliability based on the performance measurement is surprising. A direct conclusion from such an observation is that participants in our study arguably did not represent an “objective ideal observer,” which many studies have proposed as a general mechanism of sensory cue combination (Blake et al., 1993; Ernst and Bülthoff, 2004; Landy et al., 2011). Opposed to that, Knill and Saunders (2003) introduced the concept of a “subjective ideal observer” who behaves optimally according to subjective certainty. To test such an assumption, we analyzed the subjective strategy use during the presence of both signals (bimodal condition). Interestingly, most subjects claimed to have used only the belt's signal. In that sense, subjects did not behave optimally with respect to external measurements of reliability, but indeed behaved optimally with respect to the internal subjective rating of the signal's reliability. The signal that was rated to be more relevant in the unimodal conditions was used with a higher probability in the bimodal condition.

Nevertheless, the question remains as to why subjective and objective reliability measurements differ in the first place and why the subjective reliability led to increased behavioral prediction accuracy for the bimodal task. One idea would be to look at how easily and precisely the reliabilities of the two modalities can be estimated. In particular, we assume that it is advantageous to use information which is less reliable compared to information with unknown or almost-unknown reliability (no prior), even though in the end the latter might turn out to have been more reliable. In this respect, a signal's reliability might be positively biased if it can be estimated easily and quickly. On the other hand, if a signal's reliability is difficult or time-consuming to estimate (e.g., due to the lack of feedback), it might be underestimated. We argue that the belt's reliability was relatively easy to estimate for the subjects as it provides information in an absolute coordinate system and it dominated perception according to the subjective reports (as opposed to the native condition). In contrast, the reliability of the rotation information might have been quite difficult to estimate, as the vestibular system needs to integrate information over time without an absolute reference point (Barnett-Cowan and Harris, 2009). As a result, participants might have overestimated the belt's reliability and underestimated reliability based on rotation information. If such a hypothesis holds, we believe that it can have significant consequences for research investigating cue combination mechanisms and multisensory processes.

## Author contributions

CG and SP designed the experiment, recorded the participants, and analyzed the data. CG wrote the main part of the manuscript, but was supported by HF in conducting and describing the model comparisons and other mathematical procedures. HF was also responsible for the technical setup and programmed the necessary code for both the belt and the rotating platform. PK supervised the study, corrected and edited the manuscript, and suggested the way the data should be analyzed.

### Conflict of interest statement

The authors declare that the research was conducted in the absence of any commercial or financial relationships that could be construed as a potential conflict of interest.
