# Supplementary material for: Bayesian Alternation during Tactile Augmentation
Source: Front Behav Neurosci. 2016 Oct 7;10:187. doi: 10.3389/fnbeh.2016.00187 (PMC5054009; doi:10.3389/fnbeh.2016.00187)
Supplement: Supplementary file 1 [file DataSheet1.docx]

# Appendix:

## 1 Analysis of Variance for Learning Effects

**Augmented condition**

| Source | Type III Sum of Squares | df | Mean Square | F | Sig. | Partial Eta² |
| --- | --- | --- | --- | --- | --- | --- |
| Corrected Model | 154,286^a^ | 5 | 30,857 | ,347 | ,883 | ,011 |
| Intercept | 972192,857 | 1 | 972192,857 | 10945,639 | ,000 | ,985 |
| Session | 19,000 | 2 | 9,500 | ,107 | ,899 | ,001 |
| Half | 38,095 | 1 | 38,095 | ,429 | ,513 | ,003 |
| Session * Half | 97,190 | 2 | 48,595 | ,547 | ,580 | ,007 |
| Error | 14388,857 | 162 | 88,820 |  |  |  |
| Total | 986736,000 | 168 |  |  |  |  |
| Corrected Total | 14543,143 | 167 |  |  |  |  |

**Native condition**

| Source | Type III Sum of Squares | df | Mean Square | F | Sig. | Partial Eta² |
| --- | --- | --- | --- | --- | --- | --- |
| Corrected Model | 77,643^a^ | 5 | 15,529 | ,212 | ,957 | ,007 |
| Intercept | 1093517,357 | 1 | 1093517,357 | 14940,526 | ,000 | ,989 |
| Session | 32,714 | 2 | 16,357 | ,223 | ,800 | ,003 |
| Half | 36,214 | 1 | 36,214 | ,495 | ,483 | ,003 |
| Session * Half | 8,714 | 2 | 4,357 | ,060 | ,942 | ,001 |
| Error | 11857,000 | 162 | 73,191 |  |  |  |
| Total | 1105452,000 | 168 |  |  |  |  |
| Corrected Total | 11934,643 | 167 |  |  |  |  |

**Bimodal condition**

| Source | Type III Sum of Squares | df | Mean Square | F | Sig. | Partial Eta² |
| --- | --- | --- | --- | --- | --- | --- |
| Corrected Model | 81,262^a^ | 5 | 16,252 | ,242 | ,943 | ,007 |
| Intercept | 1056402,881 | 1 | 1056402,881 | 15729,735 | ,000 | ,990 |
| Session | 60,333 | 2 | 30,167 | ,449 | ,639 | ,006 |
| Half | 8,595 | 1 | 8,595 | ,128 | ,721 | ,001 |
| Session * Half | 12,333 | 2 | 6,167 | ,092 | ,912 | ,001 |
| Error | 10879,857 | 162 | 67,160 |  |  |  |
| Total | 1067364,000 | 168 |  |  |  |  |
| Corrected Total | 10961,119 | 167 |  |  |  |  |

## **
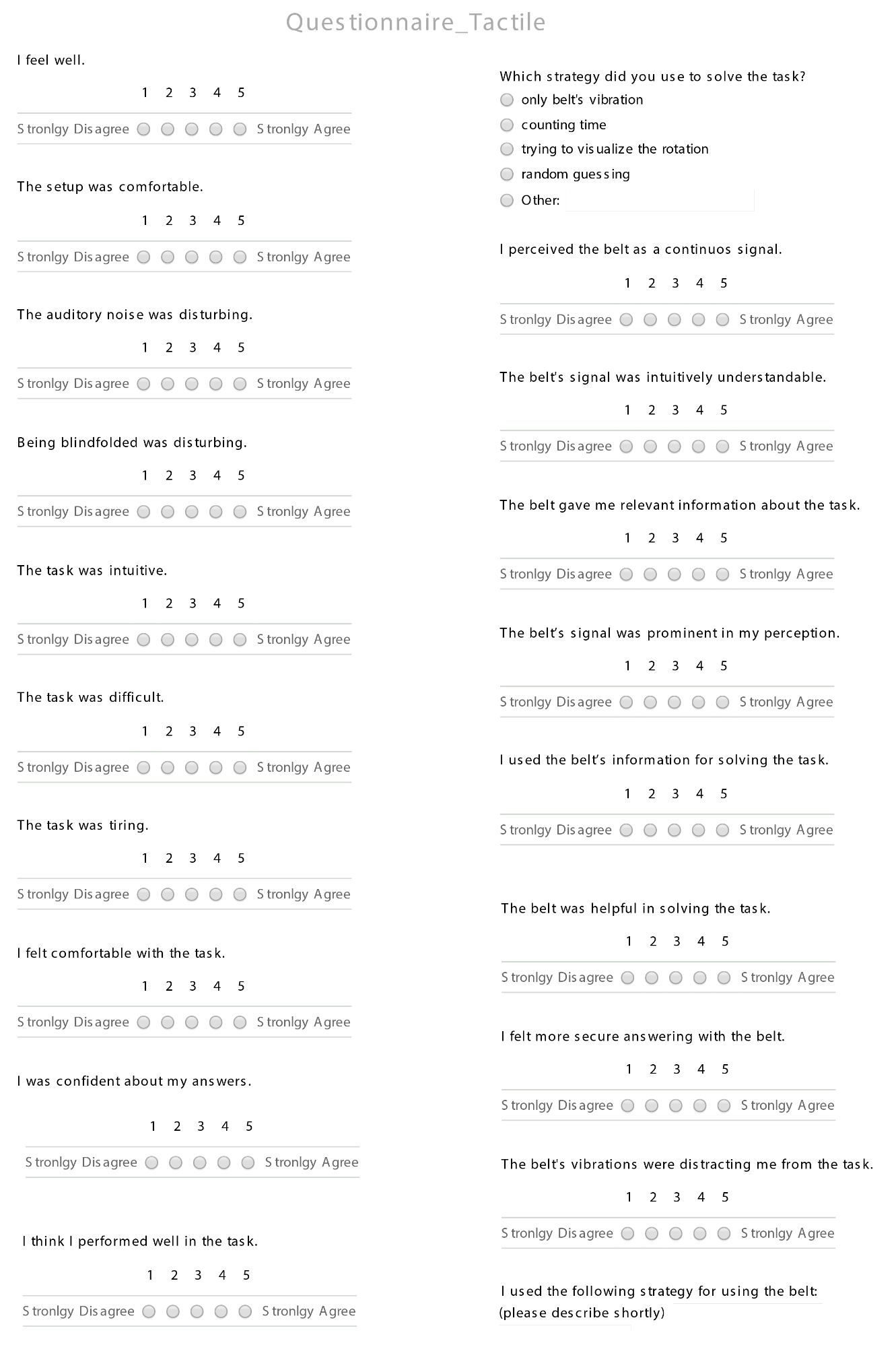
**9.2. Questionnaires

**Questionnaire Augmented Condition**

**
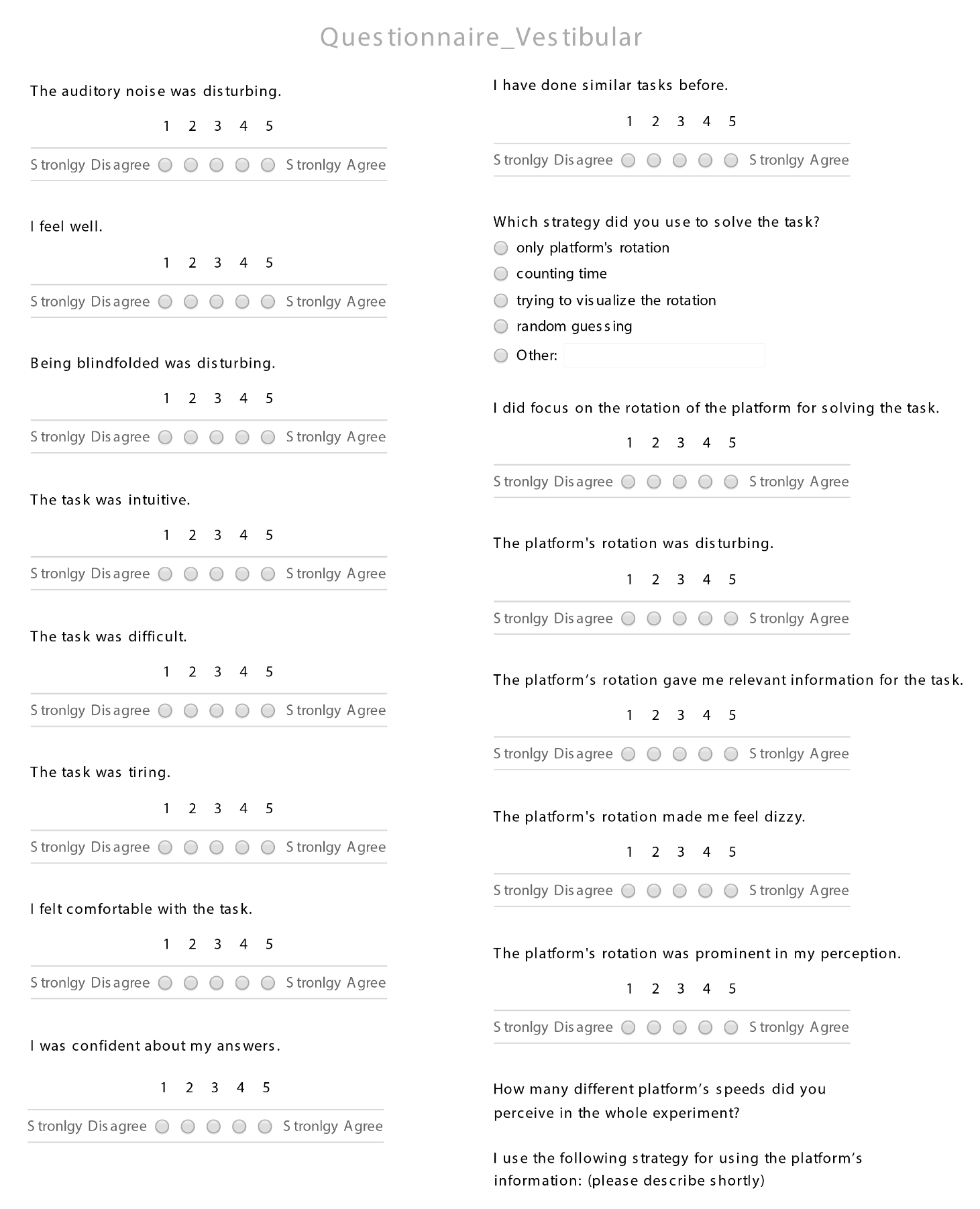
Questionnaire Native Condition**

**Questionnaire Bimodal Condition**

**
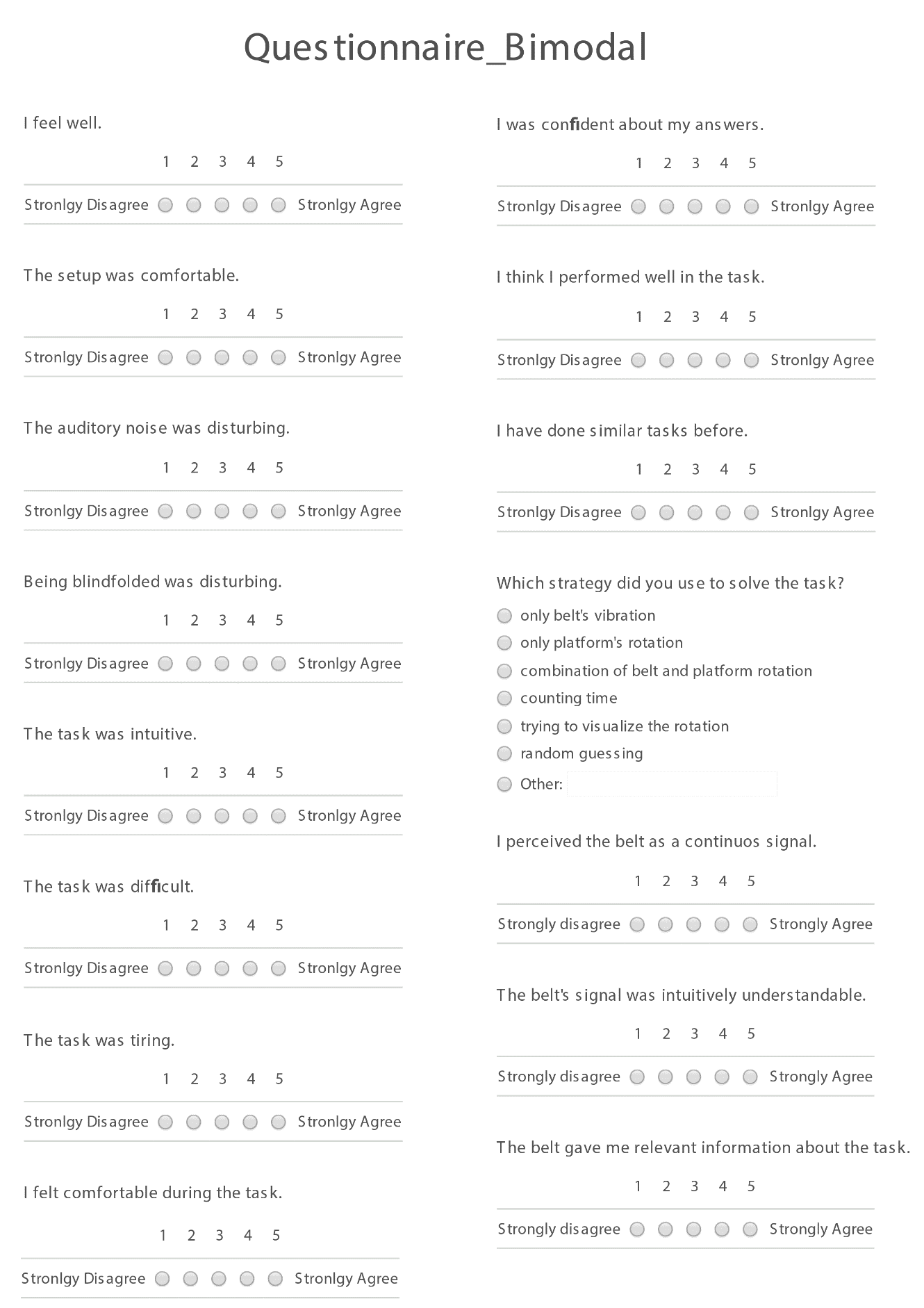
**

**
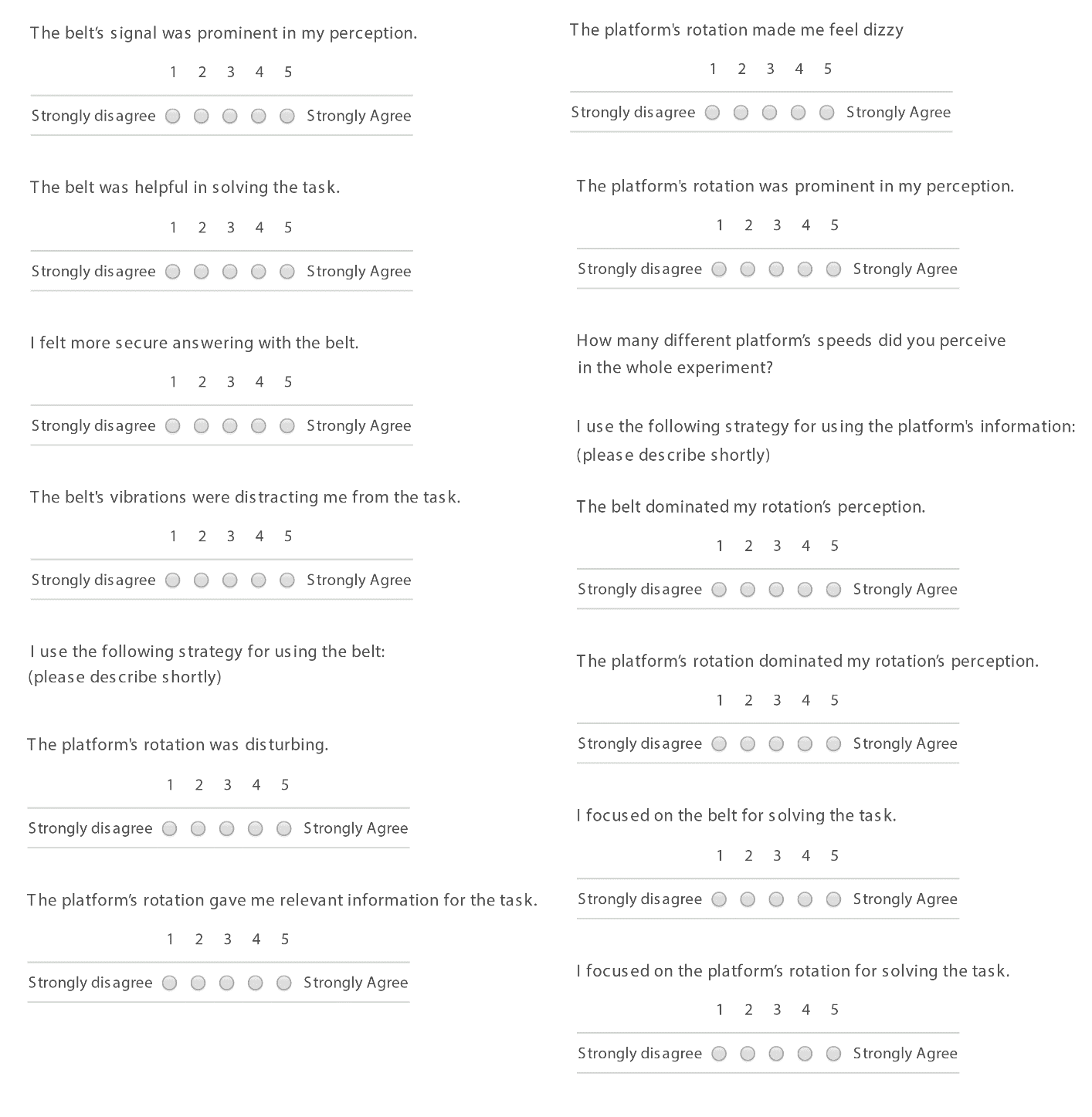
**
